# Isoprenaline: A Potential Contributor in Sick Sinus Syndrome—Insights from a Mathematical Model of the Rabbit Sinoatrial Node

**DOI:** 10.1155/2014/540496

**Published:** 2014-01-21

**Authors:** Xiang Li, Ji-qian Zhang, Jian-wei Shuai

**Affiliations:** ^1^Department of Physics and Institute of Theoretical Physics and Astrophysics, Xiamen University, Xiamen 361005, China; ^2^College of Physics and Electronic Information, Anhui Normal University, Wuhu 241000, China; ^3^Biological Physics Group, School of Physics and Astronomy, The University of Manchester, Manchester M139PL, UK

## Abstract

The mechanism of isoprenaline exerting its effects on cardiac pacemaking and driving in sick sinus syndrome is controversial and unresolved. In this paper, mathematical models for rabbit sinoatrial node cells were modified by incorporating equations for the known dose-dependent actions of isoprenaline on various ionic channel currents, the intracellular Ca^2+^ transient, and *i*_Na_ changes induced by SCN5A gene mutations; the cell models were also incorporated into an intact SAN-atrium model of the rabbit heart that is based on both heterogeneities of the SAN electrophysiology and histological structure. Our results show that, in both central and peripheral cell models, isoprenaline could not only shorten the action potential duration, but also increase the amplitude of action potential. The mutation impaired the SAN pacemaking. Simulated vagal nerve activity amplified the bradycardic effects of the mutation. However, in tissue case, the pacemaker activity may show temporal, spatial, or even spatiotemporal cessation caused by the mutation. Addition of isoprenaline could significantly diminish the bradycardic effect of the mutation and the SAN could restart pacing and driving the surrounding tissue. Positive effects of isoprenaline may primarily be attributable to an increase in *i*_Na_ and *i*_Ca,*T*_ which were reduced by the mutation.

## 1. Introduction

Sick sinus syndrome (SSS) comprises a variety of conditions involving sinus node dysfunction (SND) which occurs as a result of anatomical damage to the sinoatrial node (SAN) of the heart. Abnormalities encompassed in this syndrome include sinus bradycardia, sinus arrest or exit block, combinations of sinoatrial and atrioventricular nodal conduction disturbances, and atrial tachyarrhythmias [1]. Patients may experience syncope, presyncope, palpitations, or dizziness who have to be fitted with an electronic pacemaker [1, 2]. While the syndrome can occur in elderly and pediatric patients, it can also occur in healthy people without any evident structural heart disease but with genetic defects. Recent studies have shown that familial SSS is linked to loss-of-function mutations of the SCN5A gene [3], which result in decreased inward Na^+^ current [4, 5].

Currently, no medications are routinely used to treat symptomatic SND. However, acute treatment with the anticholinergic agent atropine and the adrenergic agonist isoprenaline (Iso) may be warranted. Studies have shown that *β*-adrenergic stimulation could increase the heart rate through accelerating the spontaneous activity of the pacemaker of the heart [6]. It is also believed that this occurs through *β*-adrenoceptor mediated modulation of ionic currents that contribute to pacemaker activity [7]. Iso is a sympathomimetic *β*-adrenergic agonist medication and known to accelerate the sinus node and enhance AV nodal conduction [8]. Experiments have shown that Iso increases the *L*-type calcium current [9–11] and the delayed rectifier potassium current [10, 11] and shifts voltage-dependent *i*_*f*_ activation curve to more positive potentials [12–15]. Furthermore, a number of clinical studies have shown that Iso exerts positive inotropic and chronotropic effects on the heart.

Whereas, to our knowledge, many researchers have investigated the effect of Iso mainly at the cellular level, its effect on the abnormal activity at the tissue level has been rarely studied before, particularly for the abnormal activity associated with sick sinus syndrome. Thus, an intriguing problem is whether patients with SSS could recover with the help of Iso and do not need cardiac pacing. In this study, we modified models of SAN to investigate theoretically the effect of Iso on the SSS and discussed the inherent mechanism between them.

## 2. Methods

### 2.1. Model Description

**Figure 1:**
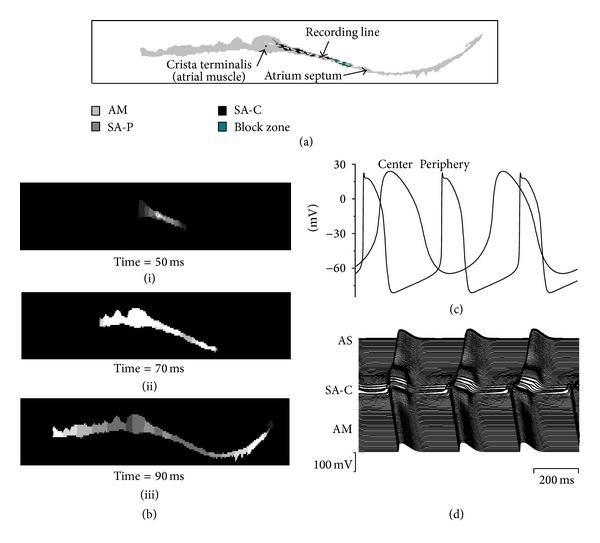
Model of the SAN and surrounding atrial tissue. (a) Color-coded distribution of cell types throughout the 2D tissue slice. (b) Snapshots of initiation and conduction in 2D anatomical model at various times after initial configuration; (i) 50 ms; (ii) 70 ms; (iii) 90 ms. (c) Action potentials in one cell of the central and peripheral SAN. (d) Action potential profiles during conduction through the slice. As, atrial septum; SA-C, SAN center; AM, atrial muscle (CT).

In order to address the above issues, mathematical model of the rabbit central and peripheral SAN cells proposed by Kurata et al. [16] was used in this paper. The action potentials generated by using the models of a central cell and a peripheral cell were shown in Figure 1(c). Besides, a 2D anatomic model of the intact SAN-atrium tissue developed by Butters et al. [17] was also used to study the effects of Iso on SAN pacemaking and driving. This 2D intact SAN-atrium tissue model was based on histologically reconstructed geometry of a single slice of the rabbit right atrium, which was cut through the atrial muscle of the crista terminalis (CT) and the intercaval region with central and peripheral SAN areas. And an experimentally observed [18, 19] nonconductive region (block zone) next to the SAN toward the atrial septum was also incorporated in this model (see Figure 1(a)). Figure 1(b) shows the initiation and conduction of pacemaker activity computed from the 2D model. Snapshots of membrane potentials across the 2D lattice were shown at 50 ms (Figure 1(b)(i)), 70 ms (Figure 1(b)(ii)), and 90 ms (Figure 1(b)(iii)) after the initial conduction. The pacemaking action was first initiated in the center (Figure 1(b)(i)) and then propagated toward the surrounding tissues (Figure 1(b)(iii)).

The mechanism underlying the genesis of SND (clinically known as SSS) is believed to be associated with changes in the intrinsic properties of the SAN with age [20]. Intercellular electrical coupling strength (*D*) is considered to reflect the changes in the intrinsic properties of the SAN. To incorporate the effects of SCN5A mutation and acetylcholine (Ach) into the models, (i) parameters of the Na^+^ current were changed according to the study of Butters et al. [17] and (ii) an Ach-activated K^+^ current (*i*_K,Ach_), the inhibited *L*-type Ca^2+^ current (*i*_Ca,*L*_), and the shift in hyperpolarization-activated current (*i*_*f*_) activation curve were introduced.

The model equations used for the cells, 2D tissue, modified Na^+^ channel current, and the channel currents relative to Ach are presented in Table 1. A detailed description of parameters of these models are listed in Table 3 and can be found in [16, 19–21].

### 2.2. Effects of Iso on Ionic Currents

In order to simulate the chronotropic effect of Iso on the pacemaker activity, Zhang et al. have incorporated a set of equations of the known actions of Iso on related ion channels into two established action potential models of rabbit SAN recently [22]. According to the study of Zhang et al., we modified the models mentioned above as follows.

1. Based on the voltage clamp experimental data on the kinetics of ionic channels and regional differences in the ionic current densities, we incorporated equations for concentration-dependent actions of Iso on the macroscopic conductance of *i*_Ca,*L*_, *i*_K,*r*_, *i*_st_, and *i*_K,*s*_ and to describe changes in the kinetics of *i*_*f*_ and *i*_K,*r*_ by the same amount as observed experimentally [23–25].
2. Because Iso was also found to increase the amplitude and minimal diastolic level of [Ca^2+^]_*i*_ in mammalian [26, 27] pacemaker cells and to alter SR Ca^2+^ uptake and release by stimulation of calmodulin kinase II [26, 28], in our simulations, we adopted the approach of Kharche et al. [29] to modify the Ca^2+^ handling equations to increase the amplitude and minimal diastolic level of [Ca^2+^]_*i*_, as observed in experimental studies [30, 31] by increasing the maximal SR Ca^2+^ release (by 20%) and reducing the SR Ca^2+^ release (by 20%) fluxes.

**Figure 2:**
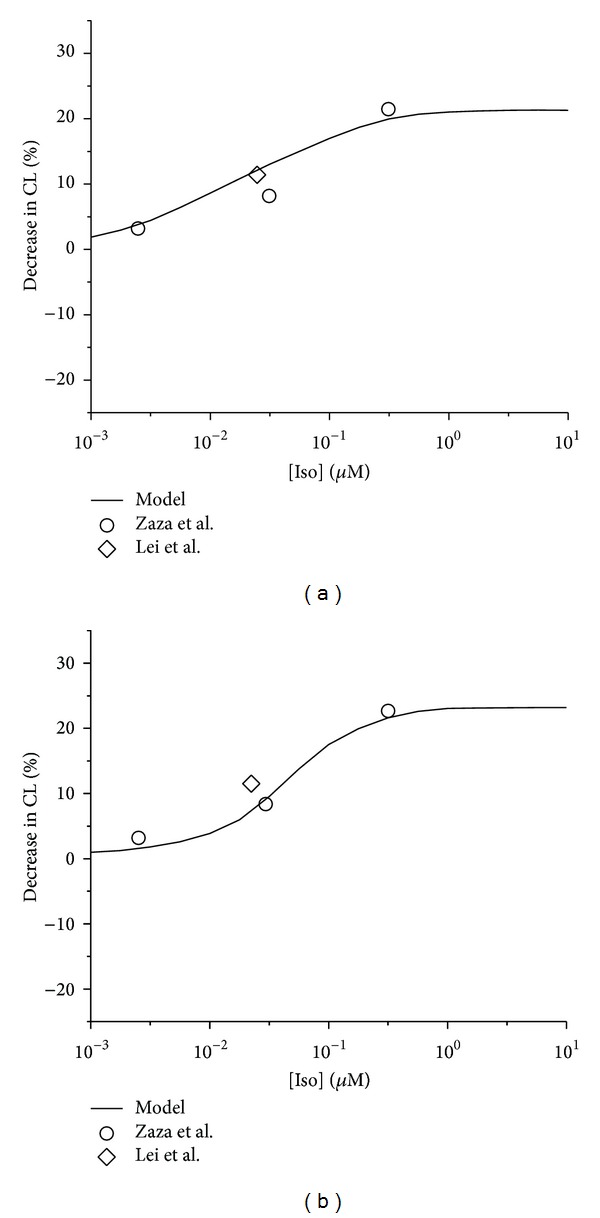
Concentration-dependent percentage decrease of pacemaking CL by Iso. (a) Central cell model and relevant experimental data. (b) Peripheral cell model and relevant experimental data. Open circles: experimental data of Zaza et al. from rabbit SAN cells. Open diamonds: data of Lei et al. from rabbit SAN cells.

A general remodeling of these ionic channels is given in Table 2. Using the models, the simulated effects of Iso are shown in Figure 2, in which the percentage of decrease of pacemaking cycle length (CL) is plotted against Iso concentration. The simulated data (solid line) from the central (Figure 2(a)) and the peripheral (Figure 2(b)) cell models were compared with the experimental data obtained from rabbit isolated SAN cells by Zaza et al. [23] (open circle) and Lei et al. [24, 25] (open diamond).

## 3. Results and Discussion

### 3.1. Iso Effects on SAN Cells

**Figure 3:**
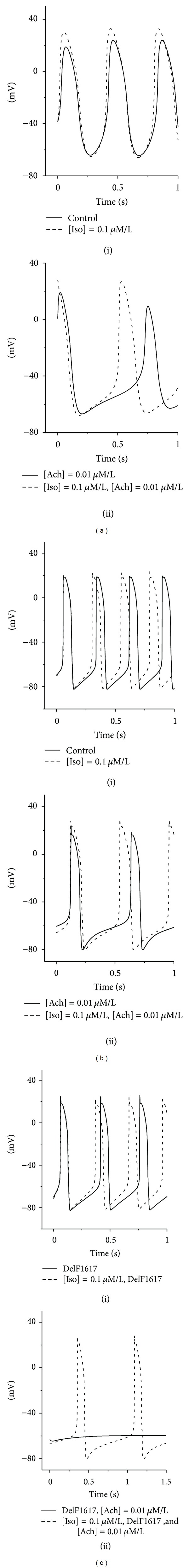
Effect of Iso on the SAN pacemaking rate. Simulated voltage from (a) central cell models and ((b) and (c)) peripheral cell models. Action potentials are shown under control conditions and in the presence 0.1 *μ*M/L Iso, 0.01 *μ*M/L Ach, and the DelF1617 mutant channel.

We discussed the effects of Iso on the dynamics of action potential in cells from the modified model. The simulated results of the action potentials under control conditions (i.e., in the absence of Iso) and in the presence of Iso (i.e., [Iso] = 0.1 *μ*M/L) are depicted in Figure 3. One can find that, with both the central and peripheral cell models, Iso shortened the action potential duration, increased the amplitude of action potentials, and caused an increase in the rate of spontaneous action potentials (Figures 3(a)(i) and 3(b)(i), dashed line). These results are consistent with experimental observations on rabbit SAN cells [23, 24].

And then, we introduced the effect of Ach into the modified models. Application of Ach slowed down pacemaking rates in both central and peripheral SAN cells, with larger effect on the central cells [32]. Simulating effects of 0.01 *μ*M/L Ach resulted in the CL increasing from 406 ms to 660 ms for the central SAN cell (Figure 3(a)(ii), solid line) and from 280 ms to 460 ms for the peripheral ones (Figure 3(b)(ii), solid line). When Iso was added (i.e, [Iso] = 0.1 *μ*M/L), the negative chronotropic effect of Ach was diminished for both types of cell models (Figures 3(a)(ii) and 3(b)(ii), dashed line).

It has been shown that the mutations slowed down pacemaking rate in peripheral, but not in central, SAN cells that control the heart rhythm [17]. Thus, we simulated the effect of Iso on pacemaking action potentials with the DelF1617 mutant channel for the peripheral SAN cells, and the results are shown in Figures 3(c)(i) and 3(c)(ii). One notices that, when only the effects of the DelF1617 mutation were introduced into the peripheral cells, the CL increased from 280 ms to 344 ms (Figure 3(c)(i), solid line). When Iso was added and its concentration was adjusted to be 0.1 *μ*M/L, the CL decreased by 14.2% (Figure 3(c)(i), dashed line). Furthermore, the negative chronotropic effect of Ach (concentration is set to be 0.01 *μ*M/L) was amplified by the mutation; that is, the cell with the wild type channel exhibited pacemaking activity (Figure 3(b)(ii), solid line), but the cell with the mutant channel became quiescent (Figure 3(c)(ii), solid line), and in this case, addition of Iso (i.e., [Iso] = 0.1 *μ*M/L) could recover the pacemaking activity of the cell with the mutant channel (Figure 3(c)(ii), dashed line). The above adjustive behavior of Iso is similar to the clinical behavior observed in patients with a longer CL [33].

### 3.2. Iso Effects on Two-Dimensional Tissues

**Figure 4:**
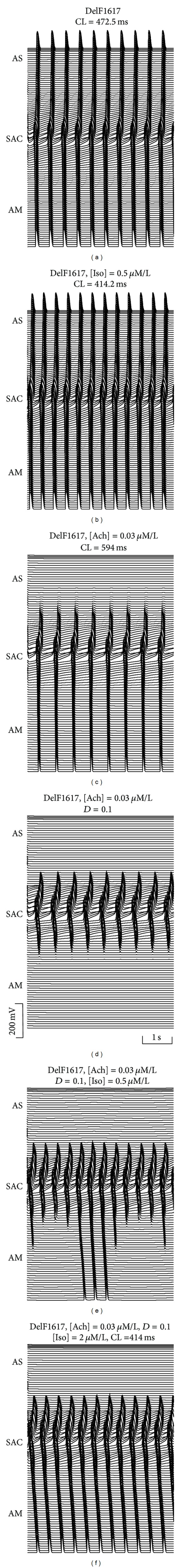
Effects of Iso on action potential conduction. Action potential profiles in the 2D tissue with the DelF1617 mutant channel under different conditions are shown. (a) Mutant channel alone; (b) [Iso] = 0.5 *μ*M/L; (c) [Ach] = 0.03 *μ*M/L; (d) [Ach] = 0.03 *μ*M/L and *D* = 0.1; (e) [Ach] = 0.03 *μ*M/L, *D* = 0.1, and [Iso] = 0.5 *μ*M/L; (f) [Ach] = 0.03 *μ*M/L, *D* = 0.1, and [Iso] = 2.0 *μ*M/L.

We investigated further the functional influences of Iso on action potential conduction across the intact SAN-atrium under control and other conditions associated with the symptoms of SSS (i.e., DelF1617 mutation; [Ach] = 0.03 *μ*M/L; *D* = 0.1 mm^2^/ms). Results are shown in Figure 4, which presents the spatial and temporal profiles of action potentials recorded from representative cells across the SAN-atrium in the model (Figure 1(a)). And the conduction in the normal tissue (with the wild type channel, *D* = 1.0 mm^2^/ms, no Iso and no Ach) is presented in Figure 1(d).

Figure 3(a) shows the action potential profile with the DelF1617 mutation. The CL was increased by 25.8% compared with the normal tissue, and the ability of the SAN to drive atrium was not impaired. However, with Ach (i.e., [Ach] = 0.03 *μ*M/L), the mutation significantly increased the CL (Figure 4(c)) and produced a conduction block in the direction toward the atrial septum; the action potential conduction in the direction toward the CT was sustained. As we know, electrotonic interaction between the SAN and the surrounding tissues played an important role in determining the SAN pacing and driving. When the coupling strength *D* was fixed to be small (i.e., *D* = 0.1 mm^2^/ms), the SAN was able to generate spontaneous activity but failed to drive the surrounding atrium; conduction exit block occurred in both directions (Figure 4(d)).

When Iso addition was simulated at tissue level, three noteworthy results can be addressed. First, pacemaking rate with the DelF1617 mutation was significantly increased. The measured CL was decreased from 472.5 ms (Figure 4(a)) to 414.2 ms (Figure 4(b)) in the presence of 0.5 *μ*M/L Iso. Second, the spatial cessation of pacemaker activity in the direction toward the atrial septum cannot be recovered by addition of Iso (Figure 4(c)). Third, a conduction block in the direction toward the CT can be avoided. Through the application of 0.5 *μ*M/L Iso, the action potential was initiated in the center and propagated toward the CT, but not continually (Figure 4(e)); however, with the Iso concentration increasing to 2.0 *μ*M/L, the spatial cessation of pacemaker activity can be recovered completely (Figure 4(f)).

**Figure 5:**
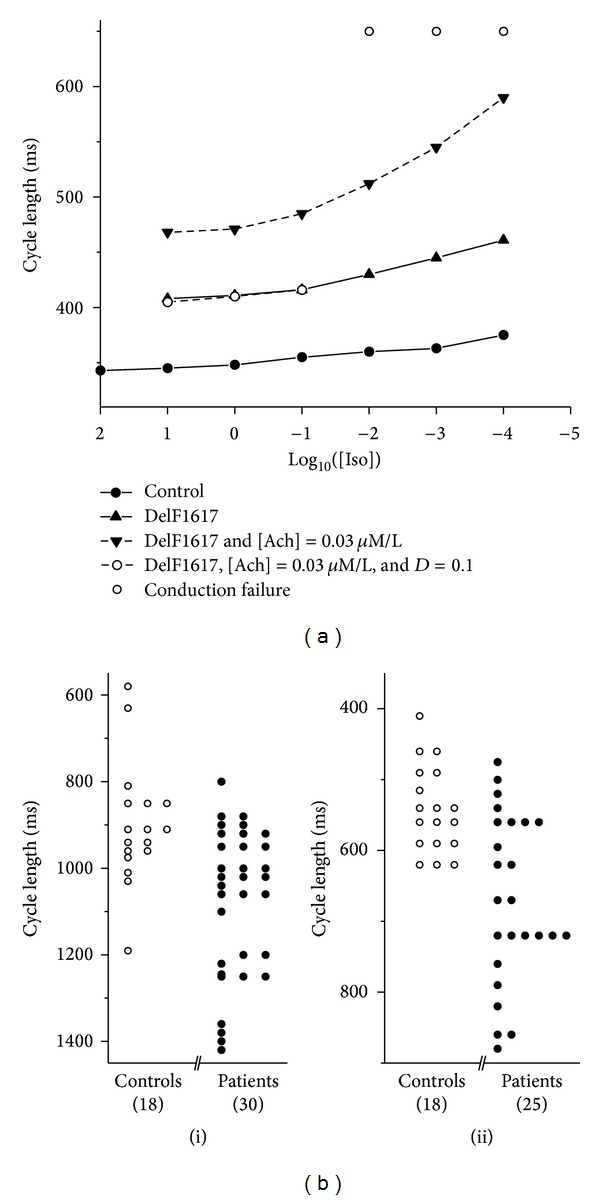
(a) Measured pacemaking CL against [Iso] computed from tissue model. Solid circles: controls; solid triangle: DelF1617 mutation; solid inverted triangles: DelF1617 and [Ach] = 0.03 *μ*M/L; open circles: DelF1617, [Ach] = 0.03 *μ*M/L, and *D* = 0.1; solid line: without conduction block; dashed line: conduction block occurs in AS. (b) Distribution of CL in controls and patients (i) before and (ii) after Iso injection. Open circles: clinical data of Vallin and Edhag from controls; solid circles: data of Vallin and Edhag from patients.

To characterize the effects of Iso, a series of simulations were performed with [Iso] changing systematically from 0.0001 *μ*M/L to 10 *μ*M/L. The results are represented in Figure 5(a), in which the computed CL was plotted against [Iso] under control conditions (solid circles), DelF1617 mutation (solid triangles), DelF1617 mutation together with [Ach] = 0.03 *μ*M/L (solid inverted triangles), and *D* = 0.1 mm^2^/ms (open circles). Under both control and DelF1617 mutation conditions, the SAN could pace and drive the atrium successfully. However, in the presence of 0.03 *μ*M/L Ach, the measured CL decreased with [Iso] in a similar way as under both control and DelF1617 mutation conditions; though the SAN paces, it does not drive the atrial septum (Figure 5(a), solid inverted triangles, dashed line).

When coupling strength was considered, another important phenomenon was that, in the range of 10^−4^ *μ*M/L ≤ [Iso] ≤ 10^−2^ *μ*M/L, the SAN failed to drive the surrounding tissues in both sides (Figure 5(a), open circles without line). When [Iso] is over 10^−1^ *μ*M/L, the SAN could drive the atrium continually (Figure 5(a), open circles, dashed line); however, conduction block in the atrial septum could not be avoided. Such transition happened because Iso has shown its positive effects on the pacemaker activity in the SAN. With [Iso] ≥ 10 *μ*M/L, the pacemaker activity of the SAN was abolished completely, which suggested that high concentrations of Iso are considered toxic. These results are consistent with clinical observations by Vallin and Edhag [33] who injected Iso in 30 patients with symptoms of sinus node disease and 18 control subjects (Figure 5(b)).

The above simulations suggested that Iso plays an important role in determining the initiation and conduction of the pacemaker activity in the SAN, especially under the conditions of mutation, high [Ach], and weak coupling strength.

### 3.3. Role of *i*_Na_ and *i*_Ca,*T*_

**Figure 6:**
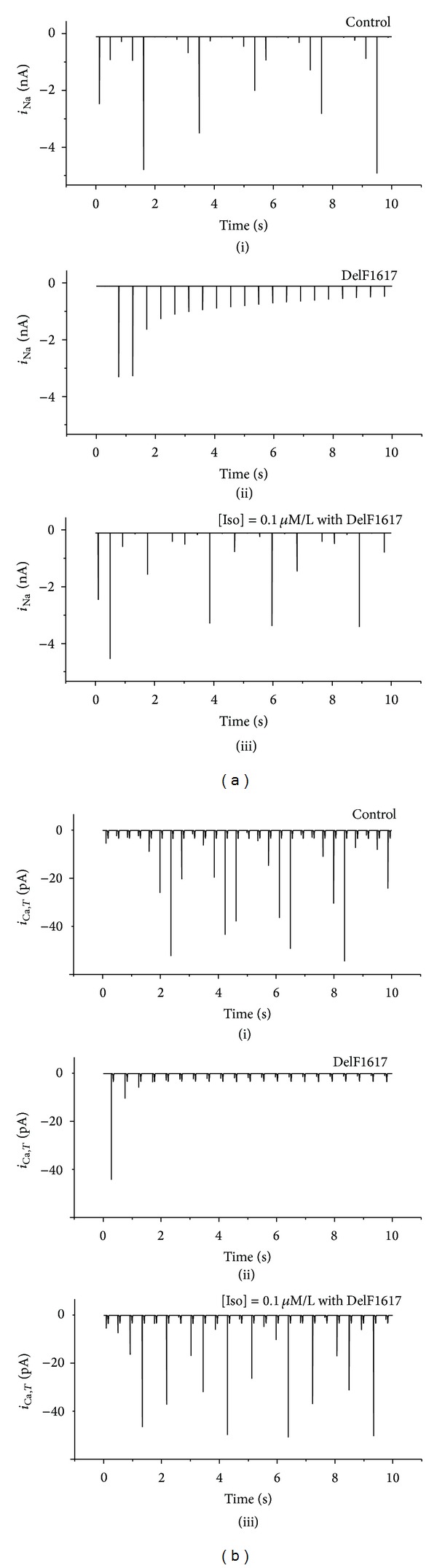
Time series of *i*_Na_ and *i*_Ca,*T*_ recording from a SAN peripheral cell under different conditions. (a) *i*_Na_; (b) *i*_Ca,*T*_. Tissues with normal conditions (i), DelF1617 mutant channel (ii), and Iso with mutant channel (iii) are shown.

Due to the functional role of several major ionic currents, it is necessary to study the corresponding deterministic kinetics of these channel currents.  Figure 6 presents the time series of the ionic currents, in which some useful clues can be found to infer the mechanism responsible for the positive effect of Iso on the pacemaker activity.

Figures 6(a)(i), (6(b)(i)), 6(a)(ii), and (6(b)(ii)) show the time series of *i*_Na_ (*i*_Ca,*T*_) under normal condition and with the DelF1617 mutation. Compared with Figures 6(a)(i) and (6(b)(i)), *i*_Na_ (*i*_Ca,*T*_) was reduced or even zero in Figures 6(a)(ii) and (6(b)(ii)). Besides, other major underlying currents (such as *i*_Ca,*L*_ and *i*_K,*r*_) were not greatly affected (data are not shown). Thus, we can conclude that the slowing of the pacemaking was primarily attributable to the mutation-induced decrease in *i*_Na_ and *i*_Ca,*T*_ (Figures 6(a)(ii) and 6(b)(ii)).

However, in this case, through the application of Iso (i.e., [Iso] = 0.1 *μ*M/L), *i*_Na_ was significantly increased as well as *i*_Ca,*T*_ (Figures 6(a)(iii) and 6(b)(iii)), and both of the currents can be even increased to a normal level. This means that proper concentration of Iso can increase *i*_Na_ and *i*_Ca,*T*_ which were reduced by the mutation. As a result of changes in the two currents, such abnormal pacemaking behavior could be eliminated.

## 4. Conclusions 

In this study, by using a 2D anatomical model of the intact SAN-atrium, we have investigated the effects of Iso and the DelF1617 mutation on initiation and conduction of the pacemaker activity. The main findings of this study are as follows. (1) Iso could accelerate the pacemaking rate and affect the shape of action potentials in both the central and peripheral SAN cells, which are consistent with experimental observations [23, 24]. (2) The DelF1617 mutation slowed down pacemaking rate. Simulated addition of Ach amplified the bradycardic effect of the mutation and compromised the ability of the SAN to pace and drive the atrial septum. (3) When combined effects of Ach and the changes in the intrinsic properties of the SAN with the mutation were considered, conduction block can occur in both directions toward the atrium and CT, leading to a higher probability of SAN exit block and sinus arrest than with the mutation or Ach alone. This finding agrees with that of Smits et al. [34] and may provide further insight into the mechanism underlying high risk of cardiac arrest in SSS patients at night, when Ach concentration is high [35, 36]. (4) Abnormal pacemaking or even cessation state can be eliminated by proper concentration of Iso. Functional effects of Iso on the other mutations were similar (i.e., T220I, P1298L, and E161 K). What is more, Iso would be also useful for the treatment of bradycardia and improve atrial mechanical function [37]. It can be seen that these results are in good accordance with the clinical findings reported by Rokseth and Hatle [27], who provided the results of a 4-year prospective study regarding the occurrence, clinical pattern, and treatment of sinus arrest in patients with acute myocardial infarction. Taken together, we conclude that Iso could improve the pacemaking ability of the SAN cells as one of the fundamental medicines for SSS.

## Figures and Tables

**Table 1:** Model equations of the SAN single cell, 2D tissue, and some channel currents.

Single cell model of SAN
*dV*/*dt* = −(*i*_Na_ + *i*_Ca,*L*_ + *i*_Ca,*T*_ + *i*_K,*r*_ + *i*_K,*s*_ + *i*_st_ + *i*_to_ + *i*_sus_ + *i*_*b*,Na_ + *i*_*b*,K_ + *i*_NaK_ + *i*_NaCa_)/*C*_*m*_
2D intact SAN-atrium tissue model
$\frac{\partial V}{\partial t}=\nabla \cdot (D\nabla V)-\frac{i_{\text{tot}}}{C_{m}}$
Changes of sodium current induced by mutation
$i_{\text{Na}}=(S_{\text{Na}}\times g_{\text{Na}})m^{3}h{\left[{\text{Na}}^{+}\right]}_{o}\frac{F^{2}}{RT}\frac{e^{(V-E_{\text{Na}})F/RT}-1}{e^{VF\text{/}RT}-1}V$
$\tau_{h_{1}}=S_{\tau_{h_{1}}}\left(\frac{3.717\times 10^{-6}e^{-0.2815(V+17.11)}}{1+3.732\times 10^{-3}e^{-0.3426(V+37.76)}}+5.977\times 10^{-4}\right)$
$\tau_{h_{2}}=S_{\tau_{h_{2}}}\left(\frac{3.186\times 10^{-8}e^{-0.6219(V+18.8)}}{1+7.189\times 10^{-5}e^{-0.6683(V+34.07)}}+3.556\times 10^{-3}\right)$
$h_{1\infty }=\frac{1}{1+e^{(V-V*S_{h}+66.1)/6.4}}$
Effects of Ach on currents
$i_{\text{K,Ach}}=g_{\text{K,Ach,max}}jk\frac{{\left[\text{Ach}\right]}^{n_{\text{K,Ach}}}}{K_{0.5,\text{K,Ach}}^{n_{\text{K,Ach}}}+{\left[\text{Ach}\right]}^{n_{\text{K,Ach}}}}\left(\frac{{\left[{\text{K}}^{+}\right]}_{o}}{10+{\left[{\text{K}}^{+}\right]}_{o}}\right)\cdot \left(\frac{V-E_{\text{K}}}{1+e^{(V-E_{\text{K}}-140)F/2.5RT}}\right)$
$i_{\text{Ca,}L}=\left(1-b_{\max}\frac{\left[\text{Ach}\right]}{K_{0.5,\text{Ca}}+[\text{Ach}]}\right)\times g_{\text{Ca},L}\left(d_{L}f_{L}+\frac{0.006}{1+e^{-(V+14.1)/6.0}}\right)\left(V-E_{\text{Ca,}L}\right)$
*i*_*f*_ = *y*[*g*_*f*,Na_(*V* − *E*_Na_) + *g*_*f*,K_(*V* − *E*_K_)]
$\frac{dy}{dt}=\frac{y_{\infty }-y}{\tau_{y}}$
$y_{\infty }=\frac{e^{-(V+78.91-s)/26.2}}{e^{-(V+78.91-s)/26.2}+e^{(V+75.131-s)/21.25}}$
$\tau_{y}=\frac{1000.0}{e^{-(V+78.91-s)/26.2}+e^{(V+75.131-s)/21.25}}$
$s=s_{\max}\frac{{\left[\text{Ach}\right]}^{n_{f}}}{K_{0.5,f}^{n_{f}}+{\left[\text{Ach}\right]}^{n_{f}}}$

**Table 2:** Actions of Iso on currents.

Increase of *L*-type Ca^2+^ current
$i_{\text{Ca,}L}=\left[\left(1+f_{\text{Ca,max}}\frac{\left[\text{Iso}\right]}{K_{0.5,{\text{Ca}}^{\prime }}+[\text{Iso}]}\right)\times g_{\text{Ca,}L}\right]\left[f_{L}d_{L}+\frac{0.006}{1+e^{-(V+14.1)/6}}\right](V-E_{\text{Ca,}L})$6
Shift of the activation curve of hyperpolarization-activated current
$y_{\infty }=\frac{e^{-(V+78.91-S_{f})/26.2}}{e^{-(V+78.91-S_{f})/26.2}+e^{(V+75.131-S_{f})/21.25}}$, $\tau_{y}=\frac{1000.0}{e^{-(V+78.91-S_{f})/26.2}+e^{(V+75.131-S_{f})/21.25}}$
$\frac{dy}{dt}=\frac{y_{\infty }-y}{\tau_{y}}$, $S_{f}=S_{f,\max}\frac{{\left[\text{Iso}\right]}^{n_{f}^{\prime }}}{K_{0.5,f^{\prime }}^{n_{f}^{\prime }}+{\left[\text{Iso}\right]}^{n_{f}^{\prime }}}$
Actions on rapid delayed rectifying K^+^ current
$i_{\text{K},r}=\left[\left(1+f_{\text{K},\max}\frac{\left[\text{Iso}\right]}{K_{0.5,g_{\text{K}}}+[\text{Iso}]}\right)\times g_{\text{K},r}\right][(1-F_{\text{K},r})p_{a,f}+F_{\text{K},r}p_{a,s}]p_{i}(V-E_{\text{K}})$
$\frac{dp_{a,f}}{dt}=\frac{1/\left(1+e^{-(V+14.2-S_{\text{K}})/10.6}\right)-p_{a,f}}{\tau_{p_{a,f}}}$, $\frac{dp_{a,s}}{dt}=\frac{1/\left(1+e^{-(V+14.2-S_{\text{K}})/10.6}\right)-p_{a,s}}{\tau_{p_{a,s}}}$
$S_{\text{K}}=S_{\text{K},\max}\frac{\left[\text{Iso}\right]}{K_{0.5,\text{Kacti}}+[\text{Iso}]}$, $\frac{dp_{i}}{dt}=\frac{1/\left(1+e^{(V+18.6)/10.1}\right)-p_{i}}{\left(1-d_{\tau_{\text{K,}r,\max}}\left(\left[\text{Iso}\right]/K_{0.5,\tau_{\text{K,}r}}+\left[\text{Iso}\right]\right)\right)\times \tau_{p_{i}}}$
Increase of slow delayed rectifying K^+^ current
$i_{\text{K},s}=\left[\left(1+f_{\text{K},\max}\frac{\left[\text{Iso}\right]}{K_{0.5,g_{\text{K}}}+\left[\text{Iso}\right]}\right)\times g_{\text{K},s}\right]x_{s}^{2}\left(V-E_{\text{K},s}\right)$
Increase of sustained inward current
$i_{\text{st}}=\left[\left(1+f_{\text{st,max}}\frac{\left[\text{Iso}\right]}{K_{0.5,\text{st}}+\left[\text{Iso}\right]}\right)\times g_{\text{st}}\right]q_{n}q_{i}\left(V-E_{\text{st}}\right)$
Actions on Ca^2+^ handling
$j_{\text{up}}=\left(1-\beta_{\text{Iso}}\right)\times P_{\text{up}}\frac{{\left({\left[\text{C}{\text{a}}^{\text{2+}}\right]}_{i}/K_{\text{mf}}\right)}^{n_{\text{up}}}-{\left({\left[\text{C}{\text{a}}^{\text{2+}}\right]}_{\text{up}}/K_{\text{mr}}\right)}^{n_{\text{up}}}}{1+{\left({\left[\text{C}{\text{a}}^{\text{2+}}\right]}_{i}/K_{\text{mf}}\right)}^{n_{\text{up}}}-{\left({\left[\text{C}{\text{a}}^{\text{2+}}\right]}_{\text{up}}/K_{\text{mr}}\right)}^{n_{\text{up}}}}$
*j*_*rel*⁡_ = (1 + α_Iso_) × *k*_*s*_ *O*([Ca^2+^]_*rel*⁡_ − [Ca^2+^]_sub_)

**Table 3:** Model parameter values.

Glossary		Central SAN model	Peripheral SAN model
*C*_*m*_	Cell membrane capacitance	20 pF	65 pF
*g*_Na_	Maximum *i*_Na_ conductance	0 *μ*S/pF	1.85 × 10^−8^ μS/pF
*g*_K,Ach,max_	Maximum *i*_K,Ach_ conductance	3.53 × 10^−10^ *μ*S/pF	1.218 × 10^−9^ μS/pF
*g*_Ca,*L*_	Maximum *i*_Ca,*L*_ conductance	2.9 × 10^−4^ *μ*S/pF	1.0 × 10^−3^ μS/pF
*g*_*f*,Na_	Maximum *i*_*f*,Na_ conductance	0.27 × 10^−4^ *μ*S/pF	1.05 × 10^−4^ μS/pF
*g*_*f*,K_	Maximum *i*_*f*,K_ conductance	0.27 × 10^−4^ *μ*S/pF	1.05 × 10^−4^ μS/pF
*g*_K,*r*_	Maximum *i*_K,*r*_ conductance	3.99 × 10^−5^ *μ*S/pF	2.46 × 10^−4^ μS/pF
*g*_*K*,*s*_	Maximum *i*_*K*,*s*_ conductance	2.59 × 10^−5^ *μ*S/pF	1.6 × 10^−4^ μS/pF
*g*_st_	Maximum *i*_st_ conductance	0.75 × 10^−5^ *μ*S/pF	0 *μ*S/pF
*E*_Ca,*L*_	Apparent reversal potential for *i*_Ca,*L*_	46.4 mV	46.4 mV
*E*_st_	Apparent reversal potential for *i*_st_	37.4 mV	37.4 mV
[Na^+^]_*o*_	Extracellular Na^+^ concentration	140 mM	140 mM
[K^+^]_*o*_	Extracellular K^+^ concentration	5.4 mM	5.4 mM
*K*_0.5,K,Ach_	[Ach] that produces a half-maximal activation of *g*_K,Ach,max_	0.28 *μ*M	0.28 *μ*M
*K*_0.5,Ca_	[Ach] that produces a half-maximal block of *i*_Ca,*L*_	0.09 *μ*M	0.09 *μ*M
*K*_0.5,Ca′_	[Iso] that produces a half-maximal increase of *i*_Ca,*L*_	7 nM	7 nM
*K*_0.5,*f*_	[Ach] that produces a half-maximal shift of *i*_*f*_ activation curve	1.26 × 10^−2^ *μ*M	1.26 × 10^−2^ *μ*M
*K*_0.5,*f*′_	[Iso] that produces a half-maximal shift of *i*_*f*_ activation curve	13.5 nM	13.5 nM
*K*_0.5,*g*_K__	[Iso] that produces a half-maximal increase of *g*_K,*r*_	19 nM	19 nM
*K*_0.5,Kacti_	[Iso] that produces a half-maximal shift of *i*_K,*r*_ activation curve	7.5 nM	7.5 nM
*K*_0.5,τ_K,*r*__	[Iso] that produces a half-maximal decrease of τ_K,*r*_	24 nM	24 nM
*K*_0.5,st_	[Iso] that produces a half-maximal Increase of *i*_st_	33 nM	33 nM
*P*_up_	Rate constant of Ca^2+^ uptake by *j*_up_ of the network SR	0.01 mM/ms	0.02 mM/ms
*n*_K,Ach_	*i*_K,Ach_ affected by [Ach] and Hill coefficient	1.5	1.5
*n*_*f*_	*i*_*f*_ affected by [Ach] and Hill coefficient	0.69	0.69
*n*_up_	SR Ca^2+^ uptake and Hill coefficient	2	2
*n*_*f*′_	*i*_*f*_ affected by [Iso] and Hill coefficient	0.392	0.392
*S*_Na_	Percentage change of *i*_Na_ conductance induced by DelF1617 mutation	0.38	0.38
*S*_τ_*h*_1___	Percentage change of the fast-inactivation time constants of *i*_Na_ induced by DelF1617 mutation	2.18	2.18
*S*_τ_*h*_2___	Percentage change of the slow-inactivation time constants of *i*_Na_ induced by DelF1617 mutation	2.75	2.75
*S*_*h*_	Parameter of the shift in the inactivation curve of *i*_Na_ induced by DelF1617 mutation	0.128	0.128
*b*_max⁡_	Maximum fraction of *i*_Ca,*L*_ block caused by Ach	0.56	0.56
*s*_max⁡_	Maximum shift of *i*_*f*_ activation curve caused by Ach	−7.2 mV	−7.2 mV
*f*_Ca,max⁡_	Maximum percentage increase of *i*_Ca,*L*_ caused by Iso	0.54	0.54
*f*_*K*,max⁡_	Maximum percentage increase of *i*_K,*r*_ caused by Iso	1.87	1.87
*f*_st.max_	Maximum percentage increase of *i*_st_ caused by Iso	1.0	1.0
*S*_*f*,max_	Maximum activation curve shift of *i*_*f*_ caused by Iso	9.62 mV	9.62 mV
*S*_K,max⁡_	Maximum activation curve shift of *i*_K,*r*_ caused by Iso	−15 mV	−15 mV
*d*_τ_K,*r,*max⁡__	Maximum decrease of τ_K,*r,*_ caused by Iso	3.0	3.0
α_Iso_	Percentage increase of the SR Ca^2+^ release fluxes caused by Iso	0.2	0.2
β_Iso_	Percentage decrease of the SR Ca^2+^ uptake caused by Iso	0.2	0.2
